# The Impact of Match Workload and International Travel on Injuries in Professional Men’s Football

**DOI:** 10.3390/sports12080212

**Published:** 2024-08-01

**Authors:** Steve den Hollander, Gino Kerkhoffs, Vincent Gouttebarge

**Affiliations:** 1 Football Players Worldwide (FIFPRO), 2132LR Hoofddorp, The Netherlands; 2 Division of Physiological Sciences, Department of Human Biology, Faculty of Health Sciences, University of Cape Town, Cape Town, Rondebosch 7701, South Africa; 3 Amsterdam UMC Location University of Amsterdam, Department of Orthopedic Surgery and Sports Medicine, Meibergdreef 9, 1105AZ Amsterdam, The Netherlands; 4 Academic Center for Evidence-Based Sports Medicine (ACES), 1105AZ Amsterdam, The Netherlands; 5 Amsterdam Movement Sciences, Aging & Vitality, Musculoskeletal Health, Sports, 1105AZ Amsterdam, The Netherlands; 6 Amsterdam Collaboration on Health & Safety in Sports (ACHSS), IOC Research Center of Excellence, 1105AZ Amsterdam, The Netherlands; 7 Section Sports Medicine, Faculty of Health, University of Pretoria, Pretoria 0028, South Africa

**Keywords:** elite, hamstring injuries, fixture congestion

## Abstract

There are concerns over the impact of the congested international match calendar on professional footballers’ physical and mental well-being, and injury susceptibility. This study aimed to determine whether there were differences in match workload and international travel between injured and non-injured male football players over two elite competition seasons. An observational, retrospective, case–control study was conducted using data from the 2021/2022 and 2022/2023 seasons of five top-tier European men’s football leagues. Student *t*-tests were used to compare cumulative match workload and international travel data over a 28-day period preceding 1270 injuries and 2540 controls. There were significant differences in match workload and international travel variables between the injured groups (all injuries and hamstring injuries) and the control group. Match workload variables were higher (*p* < 0.01), recovery variables lower (*p* < 0.01), and international travel variables higher (*p* < 0.01). An overload of match workload and international travel contribute to increased injury susceptibility in professional men’s football. This emphasizes the need to address international match calendar concerns, including the number of games per season, the frequency of back-to-back games, and international travel requirements. Additionally, the findings highlight the importance of monitoring player match workloads, and implementing squad rotations and tailored training programs to mitigate injury risks.

## 1. Introduction

In professional football, the international match calendar (IMC) is an organized schedule that directs when international and club matches are played during a season [1]. The number of games players compete in per season can vary depending on the league they play in, domestic and continental cup involvement, and their national team duties. At an elite professional level, where clubs compete for multiple domestic and continental titles, combined with national team duties, players might end up playing 70 games in a season [2]. This congestion of fixtures inevitably raises concerns about players’ physical and mental health [3]. Moreover, plans to reformat and expand major competitions, such as the UEFA Champions League, FIFA World Cup, and FIFA Club World Cup, will increase the number of matches players compete in even further [2,3].

A study of elite European club football from the 2000–2001 to 2018–2019 seasons indicated a match injury incident rate of 23.8 injuries per 1000 match-playing hours [4]. Therefore, increasing players’ match exposure will increase their susceptibility to injuries. A systematic review on the effects of fixture congestion found that injury risk increased during fixture-congested periods with fewer than four days of recovery between games [5]. In surveys of professional footballers, 35–40% reported playing too many matches per season with insufficient recovery time between games [6], and 55% of players believed they had sustained injuries due to match fixture congestion [1]. Furthermore, 65% of players felt that international travel had a negative impact on their recovery, performance, or health [6]. According to a study conducted with the Australian men’s national football team, footballers playing domestic football in Europe made an average of 9.3 nine-hour flights across multiple time zones to participate with the national team during a two-year period [7]. While an association between travel and injuries has not been established, extensive travel across multiple countries and continents is likely to have a negative impact on the performance and wellness of professional footballers [8,9]. Various intrinsic and extrinsic factors contribute to a player’s susceptibility to injury [10]. Intrinsic factors include age, technique, and previous injury, while extrinsic factors include working conditions, access to facilities, playing surface, travel, and workload. Identifying and addressing these contributing factors is crucial to reduce injuries and safeguard player welfare [10,11].

To identify and address the issue of excessive workload and to protect player welfare, FIFPRO (The Fédération Internationale des Associations de Footballeurs Professionnels) launched the Player Workload Monitoring (PWM) tool, which is a digital platform that tracks match schedules and player workload [12]. This tool focuses on three extrinsic contributing factors: match workload, rest, and international travel [12]. Currently, the PWM tool contains data from 1500 professional male footballers from the 2017–2018 season onwards [12]. Workload refers to the cumulative stress athletes endure over a given period [13]. It can be measured over acute periods (typically seven days) or chronic periods (typically 28 days) [14]. Match workload is typically quantified by the number of matches or minutes played in a given time frame [13] but can also be quantified by measuring the distance run in a match, or calculating certain actions like sprints or passes made in a game [15]. In elite men’s football, an acute overload of matches (fixture congestion) has been associated with an increased susceptibility to injury [5,13]. Conversely, an acute underload of match exposure has been associated with an increased susceptibility to muscle injuries [16], in particular, hamstring injuries [17]. As hamstring injuries constitute 24% of all injuries in elite men’s professional football [18], understanding the impact of an underload of match workload and international travel on injury susceptibility, as well as an overload, is vital to safeguard players at risk.

Therefore, the aim of this study was to determine whether there were differences in match workload (underload and overload) and international travel between injured and non-injured male football players over two seasons of elite competition. Given the high prevalence of hamstring injuries in men’s football, the study also aimed to identify differences in match workload (underload and overload) and international travel between elite male football players who sustained hamstring injuries and non-injured matched controls over the same two seasons of competition. The null hypothesis was that there were no significant differences in match workload and international travel between injured (all injuries and hamstring injuries) and non-injured elite male football players.

## 2. Materials and Methods

An observational case–control study was conducted over two football seasons (2021–2022 and 2022–2023). We used the STROBE case–control checklist when writing this paper [19].

The participants were professional male football players. The inclusion criteria were as follows:

1. The players competed in one of the following five national leagues (top five UEFA association club coefficients) [20] during the 2021/2022 and 2022/2023 football seasons:
  - Premier League (United Kingdom)
  - La Liga (Spain)
  - Serie A (Italy)
  - Bundesliga (Germany)
  - Ligue 1 (France)
2. The players were embedded within the FIFPRO PWM tool during the 2021/2022 and 2022/2023 football seasons.

Players who sustained injuries during either of the two seasons formed the injury group. The injured players were randomly matched with two players who met the inclusion criteria and who did not sustain an injury in the season in which the injured player’s injury occurred to form the control group. Controls were randomly allocated to injured players to avoid bias. A sample size calculation based on the minutes played between an injury and non-injury group in a prior study [16] indicated that 58 injury events and 116 non-injury events were required (i.e., a total sample of n = 174 to ensure a 1:2 case–control ratio) [21] to achieve a power of 80% and a level of significance of 5% (two-sided) for detecting a true difference in means between injury and non-injury groups [22].

Match workload for the 2021/2022 and 2022/2023 seasons was collected from the FIFPRO PWM tool, which is a digital platform that tracks the match workload for professional football players worldwide (https://fifpro.org/en/workload-monitoring-tool/ (accessed on 11 June 2024)). The FIFPRO PWM sources match load data from Wyscout (https://wyscout.hudl.com/ (accessed on 11 June 2024)). Wyscout’s data collection procedure, including quality controls, have been published elsewhere [23]. The following match workload variables were collected:

- Number of minutes played (club domestic league, club domestic cup, club international cup, club friendlies, national team competition, and national team friendlies) (min);
- Number of match appearances (club domestic league, club domestic cup, club international cup, club friendlies, national team competition, and national team friendlies) (n);
- Number of starting appearance (across the same match types) (n);
- Instances as an unused substitute, which is defined as the number of appearances on the bench without minutes played (across the same match types) (n);
- Instances of fewer than 3 days between appearances (n), which is a match fixture congestion cycle associated with a higher risk of injury [24];
- Instances of fewer than 5 days between appearances (n), which is a match fixture congestion cycle associated with a higher risk of injury [24];
- Number of critical zone matches, which is defined as an instance of feewer than 5 days between appearances, with a minimum of 45 min played in each appearance (n) [12].

Data on players’ international travel for their match fixtures (club and national team) during the 2021/2022 and 2022/2023 seasons were also collected from the FIFPRO PWM tool (https://fifpro.org/en/workload-monitoring-tool/ (accessed on 11 June 2024)). The following variables were collected:

- Hours spent flying (h);
- Kilometers traveled (km);
- Number of time zones crossed (n).

Injury data for the 2021/2022 and 2022/2023 seasons were collected from a publicly available data source (https://www.transfermarkt.de/ (accessed on 11 June 2024)). Each injury was subsequently verified through club and national team press releases, and/or social media posts by either the club, national team, or player. In cases where there was a discrepancy between injury data from the primary data source and the verified source, a third data source was identified to validate the injury data. Data on injury location, type, and severity were recorded in accordance with the consensus statement on injury definitions and data collection procedures in studies of football (soccer) injuries [25], and the football-specific extension of the International Olympic Committee consensus statement: methods for recording and reporting of epidemiological data on injury in sport 2020 [26].

For each injury, the cumulative match workload and international travel values were calculated over the 28-day period preceding the injury date. This was performed to determine chronic match workload and international travel for each respective variable for the injured player and corresponding controls over the same period [15]. If an injured player did not participate in a single match during the 28-day period, the injury data point and its matched controls were not included in the analysis.

Injury type, location, and severity were presented as frequencies and percentages. Descriptive statistics for chronic match workload and international travel were reported as means and standard deviations. The data were tested for normality using the Shapiro–Wilk normality test, and for variance using Levene’s test for equality of variances. Although the data were not normally distributed, as both sample sizes were greater than n = 50, an independent *t*-test and Welch *t*-test were used to compare chronic match workload and international travel variables between injury and control groups [27,28]. An independent *t*-test was used when equal variance was assumed and the Welch *t*-test when equal variance was not assumed [27]. The statistical analysis procedures were repeated to compare differences in chronic match workload and international travel variables between hamstring injuries and their respective case controls.

Statistical significance was set at *p* < 0.05. Hedge’s G effect size statistic (ES) was used to determine the magnitude of the differences between the groups. ESs were interpreted according to Hopkins et al. (2009) [29] as trivial (<0.2), small (0.2–0.59), moderate (06–1.19), large (1.2–1.99), very large (2.0–3.99), and extremely large (>0.4). All statistical analyses were performed in SPSS (version 28.01, IBM SPSS Statistics).

## 3. Results

### 3.1. Participants

Eight hundred and sixty elite male football players met the eligibility criteria for the study. The mean age of the players was 26 years old (Table 1). Thirty-one percent of the players competed in the Premier League, 19% in Ligue 1, 18% in the Serie A, 18% in La Liga, and 14% in the Bundesliga, in the period when the data were collected. All characteristics of the participants are presented in Table 1.

### 3.2. Injuries

Over the course of the two seasons, 585 players experienced at least one injury. One thousand four hundred and eighty-eight injuries were identified and verified across the two seasons. Thirty-three injuries could not be verified and were excluded from the analyses. The most common injury locations were the thigh (n = 496, 33%) and knee (n = 209, 14%). The most frequent types of injuries were muscle strain/rupture/tear (n = 825, 55%) and joint sprain/ligament tear (n = 300, 20%). Six hundred and thirty-eight injuries (43%) had a moderate severity (8–28 days), and 526 injuries (35%) were severe (>28 days). Twenty percent of injuries were hamstring injuries (n = 299).

### 3.3. Match Workload and International Travel and Injuries

A summary of chronic match workload and international travel of the players across the seasons is shown in Table 1. There were small and significant differences in all the variables between the injury group and control group (Table 2). The injured group made significantly more appearances (*p* < 0.001, ES = 0.4) and had significantly less rest time than the control group (*p* < 0.001, ES = −0.4).

### 3.4. Match Workload and International Travel and Hamstring Injuries

There were small and significant differences in all the variables between the hamstring injury group and matched control group (Table 3). The hamstring injury group made significantly more appearances (*p* < 0.001, ES = 0.6) and appearances in the starting eleven (*p* < 0.001, ES = 0.5) than the control group, and had more instances of fewer than 5 days between matches (*p* < 0.001, ES = 0.5) and significantly less rest time than the control group (*p* < 0.001, ES = −0.5).

## 4. Discussion

The objectives of this study were (1) to compare chronic match workload and international travel between elite male football players who sustained injuries and non-injured controls over two competitive seasons, and (2) to compare the same variables between players that sustained hamstring muscle injuries and non-injured controls over the same period. All the match workload and international travel variables included in the study differed significantly between the injured group (all injuries and hamstring injuries) and their respective control groups. Match workload variables (minutes played, appearances, appearances in starting eleven, fewer than 3 days between matches, fewer than 5 days between matches, and critical zone matches) were significantly higher, recovery variables (rest and unused substitute) significantly lower, and international travel variables (distance, time, and time zones crossed) significantly higher, suggesting that an overload of acute and chronic match workload and international travel contribute to increased injury susceptibility in professional men’s football.

Instances of fixture congestion (fewer than 3 days between matches, fewer than 5 days between matches, and critical zone matches) were higher in the injury group compared to controls. These findings align with the findings of a systematic review, concluding that overall injury risk increased during fixture-congested periods [5]. This correlation is understandable, as previous studies in team ball sports have shown that muscle recovery can last up to 72 h (3 days) post-game, with football and rugby players requiring longer recovery times than other team ball sports [30]. In a FIFPRO survey of 1055 professional footballers, 87% were in favor of limiting the number of back-to-back matches played in a row (matches played with fewer than 5 days of recovery time in between appearances) [31]. Additionally, 85% of the surveyed players believed that the limit of back-to-back matches should be set at six or fewer, while more than half thought it should be capped at three [1,31]. It is worth noting that our study examined the number of back-to-back matches in a 28-day period, rather than the total number of consecutive back-to-back matches. Thus, further research is warranted to determine the injury risk associated with varying numbers of back-to-back matches. This research could inform stakeholders to establish a limit that not only safeguards the players but also remains feasible within the confines of the increasingly congested IMC.

The injury cohort had a higher chronic match workload and less recovery time over the 28 days compared to the control group. Players exposed to higher chronic workload, characterized by frequent appearances and limited rest time, may experience greater physical and mental fatigue and reduced recovery capacity, predisposing them to injuries over time. Eighty-eight percent of coaches believe players should not play more than 55 games per season, i.e., 4.2 in a 28-day period, to give players adequate time for recovery and preparation [31]. Interestingly, this number falls below the mean number of appearances of the injured group (n = 4.6) and above the mean of the control group (n = 3.9). Furthermore, the injured group traveled for more hours, further distances, and across more time zones than the control group. International travel, amidst an already packed calendar with limited rest time, further reduces players’ recovery capacity and increases predisposition to injury. Traveling long distances over multiple time zones can result in travel fatigue and jet lag, further exacerbating players’ struggle to recover adequately and heightening their susceptibility to illness and injury [9]. These findings underscore the importance of managing players’ workload over extended periods of time to effectively mitigate injury risks. By monitoring both acute and chronic workloads, stakeholders can implement targeted interventions to optimize performance and minimize injury susceptibility. This may include implementing squad rotation strategies, scheduling adequate rest periods in the IMC, and tailoring training programs to ensure adequate rest and recovery between games. In addition, particular attention should be given to players traveling long distances over multiple time zones.

Hamstring injuries constituted 20% of all injuries, reflecting findings of the UEFA Elite Club Injury Study, where they constituted 24% of all injuries [18]. An overload of match workload was a significant contributor to hamstring injuries when compared to the control group. The UEFA Elite Club Injury Study suggested that factors such as the crowded IMC and extensive international travel may increase hamstring injury susceptibility [18], which is a hypothesis supported by the findings of our study. Contrastingly, hamstring injuries have also been associated with an underload of match workload, specifically minutes played [17]. A study by Moreno-Perez (2023) examined data from two La Liga teams across the 2011–2014 seasons [17]; variations in match exposure, high-risk activities during matches, and changes in travel variables between teams and competitions, as well as over time, could explain the differences observed in the results. However, the findings underscore the importance of managing match workload effectively to mitigate the risk of hamstring injuries, both in terms of underload and overload.

The use of publicly available data provided access to a large dataset encompassing professional male football players from multiple clubs, nations, and leagues, improving the generalizability and applicability of our findings. Furthermore, the use of the FIFPRO PWM tool ensured that match workload and international travel data were collected in a standardized manner [23,32]. However, the use of publicly available data is not without its limitations. Specifically, regarding injury data, concerns have been raised regarding the accuracy of publicly available sources [33]. To address these concerns, each injury data point was verified, and when it could not be verified, it was removed from the dataset. The mechanism of injury was not described in this study. As such, some of the injuries recorded may have been caused by an inciting event and not an accumulation of load or fatigue. While this study focused on match workload and international travel, it is important to acknowledge that various intrinsic and extrinsic factors, including playing styles, coaching strategies, and individual player characteristics, can also impact a player’s susceptibility to injury [10]. Furthermore, training data were not included in the dataset, as it is not publicly available. In future studies, it would be beneficial to include training data to offer a more complete understanding of player workload. Additionally, exploring the role of playing positions, styles, and coaching strategies could provide valuable insights into reducing injury susceptibility.

The findings of this study underscore the importance of managing players’ workload over extended periods of time to effectively mitigate injury risks. By monitoring both acute and chronic workloads, stakeholders can implement targeted interventions to optimize performance and minimize injury susceptibility. Strategies may include implementing squad rotation, scheduling adequate rest periods in the IMC, and tailoring training programs to ensure adequate rest and recovery between games. Footballers should also be granted at least one day off per week, aligning with standard practices in most industries. In addition, particular attention should be given to players traveling long distances over multiple time zones. Ideally, matches should not be scheduled in the first 48 h after national team duties.

## 5. Conclusions

In conclusion, injured players (all injuries and hamstring injuries) had higher match workloads in the 28 days leading up to their injuries compared to non-injured players. Additionally, injured players experienced a greater number of back-to-back matches in the 28 days leading up to their injuries compared to the non-injured group. Injured players also traveled further, for longer, and across more time zones than the non-injured group.

The findings highlight the importance of closely monitoring player match workloads and international travel requirements to mitigate injury risks. The findings also align with the perceptions of players regarding match workload, travel, and injury susceptibility, and address concerns regarding the IMC, including the number of games in a season, the frequency of back-to-back games, and international travel requirements.

## Figures and Tables

**Table 1:** Age, chronic match workload, and international travel variables (over a 28-day period) of all professional male football players across seasons and both seasons combined (total).

Variables	2021–2022 (n = 1743)		2022–2023 (n = 2067)		Total (n = 3810)	
	Mean	SD	Mean	SD	Mean	SD
Age	25.9	3.9	26.5	3.9	26.3	3.9
Minutes Played (min)	326	159	305	169	315	165
Appearances (n)	4.2	1.7	4.1	1.8	4.1	1.8
Appearances in Starting Eleven (n)	3.5	1.8	3.3	1.9	3.4	1.8
Unused Substitute (n)	0.6	1.0	0.7	1.2	0.6	1.1
Rest Time (min)	572	42	574	44	573	43
Fewer than 3 Days between Matches (n)	0.6	0.9	0.6	0.9	0.6	0.9
Fewer than 5 Days between Matches (n)	2.1	1.9	1.9	2.0	2.0	1.9
Critical Zone Matches (n)	1.5	1.6	1.4	1.6	1.4	1.6
Distance Traveled (km)	3234	5288	1861	4156	2489	4756
Travel Time (h)	4.5	6.9	2.6	5.5	3.5	6.3
Time Zones Crossed (n)	1.5	3.0	1.1	3.1	1.3	3.0

SD = standard deviation; min = minutes; n = number; km = kilometers; h = hours.

**Table 2:** Differences in workload variables between injury and control groups.

Workload Variables	Injury (n = 1270)		Control (n = 2540)		Injury vs. Control	
	Mean	SD	Mean	SD	ES	Interpretation
Minutes Played (min)	337	164	303	164	0.2 *	Small
Appearances (n)	4.6	1.7	3.9	1.8	0.4 *	Small
Appearances in Starting Eleven (n)	3.8	1.9	3.2	1.8	0.3 *	Small
Unused Substitute (n)	0.5	0.9	0.7	1.2	−0.2 *	Small
Rest Time (min)	562	42	578	42	−0.4 *	Small
Fewer than 3 Days between Matches (n)	0.7	0.9	0.6	0.9	0.2 *	Small
Fewer than 5 Days between Matches (n)	2.4	2.0	1.8	1.8	0.3 *	Small
Critical Zone Matches (n)	1.7	1.8	1.3	1.6	0.2 *	Small
Distance Traveled (km)	3041	5218	2213	4483	0.2 *	Small
Travel Time (h)	4.2	6.9	3.1	5.9	0.2 *	Small
Time Zones Crossed (n)	1.6	3.4	1.1	2.8	0.2 *	Small

SD = standard deviation; ES = Effect Size; * *p* < 0.05; min = minutes; n = number; km = kilometers; h = hours.

**Table 3:** Differences in workload variables between hamstring injury and control groups.

Workload Variables	Injury (n = 260)		Control (n = 520)		Injury vs. Control	
	Mean	SD	Mean	SD	ES	Interpretation
Minutes Played (min)	362	160	310	162	0.3 *	Small
Appearances (n)	4.9	1.7	4.0	1.7	0.6 *	Small
Appearances in Starting Eleven (n)	4.1	1.9	3.3	1.8	0.5 *	Small
Unused Substitute (n)	0.4	0.8	0.7	1.2	−0.2 *	Small
Rest Time (min)	554	40	577	41	−0.6 *	Small
Fewer than 3 Days between Matches (n)	0.9	1.0	0.6	0.9	0.3 *	Small
Fewer than 5 Days between Matches (n)	2.8	2.0	1.8	1.8	0.5 *	Small
Critical Zone Matches (n)	2.0	1.8	1.4	1.6	0.4 *	Small
Distance Traveled (km)	3264	5299	2075	4024	0.3 *	Small
Travel Time (h)	4.5	7.0	2.9	5.4	0.3 *	Small
Time Zones Crossed (n)	1.6	3.1	1.1	2.8	0.2 *	Trivial

SD = standard deviation; ES = Effect Size; * *p* < 0.05; min = minutes; n = number; km = kilometers; h = hours.

## Data Availability

Restrictions apply to the availability of these data. Player workload data were obtained from FIFPRO and Benchmark and are available from the authors with permission from FIFPRO and Benchmark.

## References

[1] Pillay L., Burgess D., van Rensburg D.C.J., Kerkhoffs G.M., Gouttebarge V. (2022). The congested International Match Calendar in football: Views of 1055 professional male players. BMC Sports Sci. Med. Rehabil..

[2] Dalen-Lorentsen T., Bjorneboe J., Haroy J., Andersen T.E. (2024). We stand with the players: A call to action for the football community. Br. J. Sports Med..

[3] FIFPRO (2023). Extreme calendar congestion: The adverse effects on player health and wellness. PWM Annual Workload Report.

[4] Ekstrand J., Spreco A., Bengtsson H., Bahr R. (2021). Injury rates decreased in men’s professional football: An 18-year prospective cohort study of almost 12,000 injuries sustained during 1.8 million hours of play. Br. J. Sports Med..

[5] Page R.M., Field A., Langley B., Harper L.D., Julian R. (2023). The Effects of Fixture Congestion on Injury in Professional Male Soccer: A Systematic Review. Sports Med..

[6] Gouttebarge V., Brink M.S., Kerkhoffs G.M.M.J. (2019). The perceptions of elite professional footballers on the International Match Calendar: A cross-sectional study. Sci. Med. Footb..

[7] Clements E., Ehrmann F., Clark A., Jones M., Lu D., Duffield R. (2023). The type and extent of travel for professional footballers undertaking national team duties for a national football federation. Biol. Sport.

[8] Fowler P., Duffield R., Waterson A., Vaile J. (2015). Effects of regular away travel on training loads, recovery, and injury rates in professional Australian soccer players. Int. J. Sports Physiol. Perform..

[9] Janse van Rensburg D.C., Jansen van Rensburg A., Fowler P.M., Bender A.M., Stevens D., Sullivan K.O., Fullagar H.H.K., Alonso J.M., Biggins M., Claassen-Smithers A. (2021). Managing Travel Fatigue and Jet Lag in Athletes: A Review and Consensus Statement. Sports Med..

[10] Meeuwisse W. (1994). Assessing causation in sport injury: A multifactoral model. Clin. J. Sport Med..

[11] Meeuwisse W.H., Tyreman H., Hagel B., Emery C. (2007). A dynamic model of etiology in sport injury: The recursive nature of risk and causation. Clin. J. Sport Med..

[12] FIFPRO Player Workload Monitoring—Notes & Methodology. https://fifpro.org/en/workload-monitoring-tool.

[13] Jiang Z., Hao Y., Jin N., Li Y. (2022). A Systematic Review of the Relationship between Workload and Injury Risk of Professional Male Soccer Players. Int. J. Environ. Res. Public. Health.

[14] Hulin B.T., Gabbett T.J., Lawson D.W., Caputi P., Sampson J.A. (2016). The acute:chronic workload ratio predicts injury: High chronic workload may decrease injury risk in elite rugby league players. Br. J. Sports Med..

[15] Soligard T., Schwellnus M., Alonso J.M., Bahr R., Clarsen B., Dijkstra H.P., Gabbett T., Gleeson M., Hagglund M., Hutchinson M.R. (2016). How much is too much? (Part 1) International Olympic Committee consensus statement on load in sport and risk of injury. Br. J. Sports Med..

[16] Moreno-Perez V., Paredes V., Pastor D., Garrosa F.N., Vielcazat S.J., Del Coso J., Mendez-Villanueva A. (2021). Under-exposure to official matches is associated with muscle injury incidence in professional footballers. Biol. Sport.

[17] Moreno-Perez V., Del Coso J., Lopez-Del Campo R., Resta R., Romero-Sanguesa J., Courel-Ibanez J., Mendez-Villanueva A. (2023). Reduced Match Exposure in the Previous 2 Matches Accounts for Hamstring Muscle Injury Incidence in Professional Football Players. Sports Health.

[18] Ekstrand J., Bengtsson H., Walden M., Davison M., Khan K.M., Hagglund M. (2022). Hamstring injury rates have increased during recent seasons and now constitute 24% of all injuries in men’s professional football: The UEFA Elite Club Injury Study from 2001/02 to 2021/22. Br. J. Sports Med..

[19] von Elm E., Altman D.G., Egger M., Pocock S.J., Gotzsche P.C., Vandenbroucke J.P., Initiative S. (2007). Strengthening the Reporting of Observational Studies in Epidemiology (STROBE) statement: Guidelines for reporting observational studies. BMJ.

[20] UEFA Association Club Coefficients. https://www.uefa.com/nationalassociations/uefarankings/country/?year=2023.

[21] Lewallen S., Courtright P. (1998). Epidemiology in practice: Case-control studies. Community Eye Health.

[22] Dhand N.K., Khatkar M.S. Statulator: An Online Statistical Calculator. Sample Size Calculator for Comparing Two Independent Means. http://statulator.com/SampleSize/ss2M.html.

[23] Pappalardo L., Cintia P., Rossi A., Massucco E., Ferragina P., Pedreschi D., Giannotti F. (2019). A public data set of spatio-temporal match events in soccer competitions. Sci. Data.

[24] Carling C., McCall A., Le Gall F., Dupont G. (2016). The impact of short periods of match congestion on injury risk and patterns in an elite football club. Br. J. Sports Med..

[25] Fuller C.W., Ekstrand J., Junge A., Andersen T.E., Bahr R., Dvorak J., Hagglund M., McCrory P., Meeuwisse W.H. (2006). Consensus statement on injury definitions and data collection procedures in studies of football (soccer) injuries. Br. J. Sports Med..

[26] Walden M., Mountjoy M., McCall A., Serner A., Massey A., Tol J.L., Bahr R., D’Hooghe M., Bittencourt N., Della Villa F. (2023). Football-specific extension of the IOC consensus statement: Methods for recording and reporting of epidemiological data on injury and illness in sport 2020. Br. J. Sports Med..

[27] West R.M. (2021). Best practice in statistics: Use the Welch t-test when testing the difference between two groups. Ann. Clin. Biochem..

[28] Rasch D., Teuscher F., Guiard V. (2007). How robust are tests for two independent samples?. J. Stat. Plan. Inference.

[29] Hopkins W.G., Marshall S.W., Batterham A.M., Hanin J. (2009). Progressive statistics for studies in sports medicine and exercise science. Med. Sci. Sports Exerc..

[30] Doeven S.H., Brink M.S., Kosse S.J., Lemmink K. (2018). Postmatch recovery of physical performance and biochemical markers in team ball sports: A systematic review. BMJ Open Sport Exerc. Med..

[31] FIFPRO (2022). Player and High Performance Coach Surveys.

[32] Sánchez-López R., Echeazarra I., Castellano Paulis J. (2023). Validación de un instrumento para calificar la competencia futbolística a partir de Wyscout. Apunts Educación Física y Deportes.

[33] Hoenig T., Edouard P., Krause M., Malhan D., Relogio A., Junge A., Hollander K. (2022). Analysis of more than 20,000 injuries in European professional football by using a citizen science-based approach: An opportunity for epidemiological research?. J. Sci. Med. Sport.

[34] Canadian Institutes of Health Research, Natural Sciences and Engineering Research Council of Canada, Social Sciences and Humanities Research Council of Canada (2022). Tri-Council Policy Statement Ethical Conduct for Research Involving Humans.

